# The Sound Absorption Performance of Laser-Sintered Composite Biomimetic Wood Porous Structures

**DOI:** 10.3390/polym16233290

**Published:** 2024-11-26

**Authors:** Li Zou, Aitian Zhang, Zhenbo Liu, Pengfei Du, Yanling Guo

**Affiliations:** 1 College of Materials Science and Engineering, Northeast Forestry University, Harbin 150040, China; zouli@nefu.edu.cn; 2 College of Mechanical and Electrical Engineering, Northeast Forestry University, Harbin 150040, China; dupengfei@nefu.edu.cn (P.D.); nefugyl@hotmail.com (Y.G.)

**Keywords:** wood–plastic composites, biomimetic structure, porous combination structure, sound absorption, laser sintering

## Abstract

This study investigates the development of biomimetic sound-absorbing components through laser sintering technology, drawing inspiration from wood’s natural porous structure. Using a pine wood powder/phenolic resin composite, various specimens were fabricated with different structural configurations (solid, fully porous, and varying straight-pore ratios) and cavity thicknesses. Sound absorption performance was evaluated using the impedance tube transfer function method. The effect of different composite structures, placement orientations, and cavity thicknesses on sound absorption performance was evaluated. The results demonstrate that solid laser-sintered samples exhibit inherent sound absorption properties due to microscopic pores, with absorption coefficients exceeding 0.234. The biomimetic wood-like structure, featuring multi-scale porosity at both microscopic and mesoscopic levels, shows enhanced broadband sound absorption, particularly in mid-high frequencies, with characteristic double-peak absorption curves. The study reveals that absorption performance can be optimized by adjusting structural parameters and thickness, enabling targeted frequency-specific sound absorption. This research establishes the feasibility of creating multi-frequency sound-absorbing materials using laser-sintered biomimetic wood structures, providing a foundation for future applications and development in acoustic engineering.

## 1. Introduction

The rapid industrialization, urbanization, and expansion of transportation infrastructure have precipitated a significant increase in noise pollution, a pervasive environmental concern with profound implications for both human health and ecosystem integrity [1,2]. Noise is the sound emitted when the sound-emitting body vibrates irregularly, and it is a haphazard combination of sounds of different frequencies and intensities [3]. High-frequency noise usually refers to noise signals with frequencies exceeding several kilohertz. High-frequency noise is usually generated by electromagnetic interference from electronic equipment, the vibration of high-speed mechanical equipment, etc. Low-frequency noise usually refers to noise signals with frequencies below 500 Hz. Low-frequency noise is generally generated by the operation of heavy equipment, transportation, etc. [4]. This escalating issue has catalyzed extensive research into noise mitigation strategies, with particular emphasis on the development and application of advanced sound-absorbing materials and structures [5]. Sound absorption mechanisms can be broadly categorized into two primary classes: porous materials and resonant structures. Porous acoustic materials, comprising fibers, particles, and foams, demonstrate superior efficacy in attenuating mid- and high-frequency noise. However, their performance in the low-frequency spectrum is suboptimal. Conversely, resonant acoustic structures exhibit exceptional low-frequency sound absorption capabilities [6].

Resonant acoustic structures operate on the principle of frequency-matched energy conversion. When incident sound waves align with the structure’s intrinsic resonant frequency, the resulting amplified vibration facilitates the transformation of acoustic energy into mechanical and thermal energy, effectively attenuating noise [7]. This mechanism underpins the efficacy of resonant structures in sound absorption. Contemporary resonant acoustic structures primarily encompass perforated plate, micro-perforated plate, and thin plate resonators. Perforated resonance structures, typically characterized by small or straight holes, exhibit a relatively narrow bandwidth of acoustic absorption [8]. Their efficacy is predominantly confined to the low- and mid-frequency ranges, with limited high-frequency absorption capabilities. This frequency-dependent performance inherently constrains the application scope of perforated resonance acoustic structures.

Wood, as a natural composite, exhibits a hierarchical porous structure derived from its diverse cellular composition, providing an ideal template for bionic material design [9]. Its porosity stems from anatomical features such as vessels, wood fibers (in hardwoods), tracheids (in softwoods), ray cells, and axial parenchyma [10,11]. These cavity cell structures not only give wood its unique physical properties but also provide the basis for the development of new functional materials, such as acoustic materials.

The advent of 3D printing technologies, particularly fused deposition modeling (FDM), has demonstrated significant potential in fabricating complex resonant sound-absorbing structures [12]. The increasing accessibility and versatility of 3D printing are expected to drive its adoption in acoustic applications [13]. Dong et al. [14] utilized FDM to create biomimetic sound-absorbing materials replicating wood’s internal structure. Concurrently, international research teams have made strides in developing three-dimensional materials for low-frequency broadband sound absorption, leveraging local resonance and structural modal coupling [15].

Laser sintering technology has also shown promise in acoustic material manufacturing. Liu et al. [16] evaluated the acoustic performance of additively manufactured porous polycarbonate, offering new perspectives on 3D-printed acoustic material design. Nansha and Hong [17] developed micro-spiral metamaterials via 3D printing, elucidating the significant influence of spiral vestibule and cavity depth on sound absorption coefficients. Dupont et al. [18] proposed a low-frequency sound attenuation design based on periodic resonator arrays, focusing on material microstructures.

Traditional perforated sound-absorbing structures are limited by narrow and singular effective frequency bands. To address these limitations, this study proposes a novel biomimetic wood-inspired porous composite sound-absorbing structure fabricated using selective laser sintering (SLS) of a porous green biomass composite material.

SLS, an advanced additive manufacturing technique, employs precisely controlled laser beams to melt powdered materials layer by layer, forming complex 3D structures [19]. This method offers advantages such as material versatility, high utilization efficiency [20], diverse part properties, and elimination of complex support systems [21], making it particularly suitable for fabricating intricate structures [22].

In this study, perforated panels are prepared using SLS technology by selecting suitable porous acoustic materials and designing a new bionic acoustic structure. The study of the sound absorption characteristics of the new bionic structure enables this special perforated plate to realize multi-frequency band sound absorption with wide bandwidth characteristics. This research utilizes pine powder/phenolic resin as the SLS printing medium to create a biomimetic porous structure inspired by wood’s microscopic architecture. The study investigates the acoustic performance of composite sintered parts with varying porous structures and cavity thicknesses, analyzing how structural modifications and cavity dimensions influence the sound absorption characteristics of the wood powder/phenolic sintered specimens. By employing SLS technology to fabricate this biomimetic porous structure, the research aims to overcome the limitations of traditional perforated sound-absorbing designs. The findings provide a theoretical foundation for future research and development of 3D-printed biomimetic wood porous composite structures.

## 2. Experimental Materials and Methods

### 2.1. Material Selection Analysis

The application of biomass materials in the field of acoustics has received increasing attention in recent years, mainly in the areas of sound absorption, sound insulation, and acoustic wave modulation. Biomass materials such as natural fibers (hemp, coir, wool, etc.) and wood are ideal for sound absorption applications. These materials are effective in absorbing sound energy, especially in the mid- and high-frequency ranges, due to their porous structure. Berardi et al. [23] used discarded plant fibers to make acoustic panels, which not only provide excellent acoustic performance but also reduce cost and environmental impact. In addition, mycelial composites are a new type of biomass material that forms porous structures by solidifying plant waste through fungal growth. Girometta et al. [24] showed that these materials not only have excellent thermal insulation properties but also exhibit good acoustic performance.

This study used selective laser sintering as a processing and preparation method. Selective laser sintering (SLS) technology, utilizing laser energy and powdered materials, is theoretically compatible with any material capable of laser-induced bonding, including polymers, ceramics, biomass, metals, and their composites [25]. Biomass composite materials, with their multi-scale and multi-level porous structures, create complex sound wave propagation paths, facilitating energy attenuation through multiple dissipation mechanisms. This unique acoustic property, combined with reproducibility and environmental sustainability, renders biomass composites ideal for SLS processing. The precise control afforded by SLS technology enables the optimization of material microstructures and directional manipulation of acoustic performance.

Biomass processing residues, being widely available and renewable, offer sustainable resource utilization [26]. This study employs pine wood powder, a forestry by-product, as the biomass-reinforcing phase, aligning with green design principles and promoting resource recycling [27]. Pine wood powder’s primary components—cellulose, hemicellulose, and lignin—contribute to its structural integrity, with cellulose (approximately 40% of the total composition) forming a supportive network that enhances mechanical properties [28]. Based on the advantages of pine powder, pine powder was selected as a structural material to be added to the phenolic resin matrix, and pine/phenolic resin composites were chosen as the raw material for SLS. Phenolic resin is a kind of polymer material produced through the polycondensation reaction between phenolic compounds and aldehydes. As one of the first polymer resins synthesized by mankind, it has good mechanical strength and dimensional stability.

**Figure 1:**
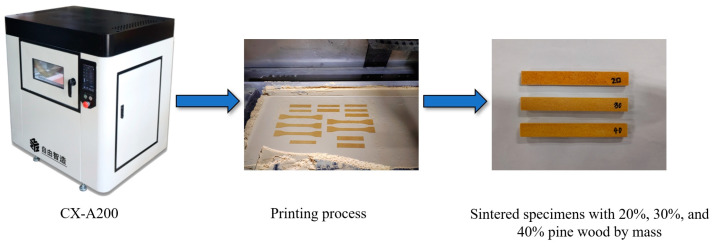
Preparation of sintered specimens with different pine wood percentages.

This study utilized mechanical blending to prepare pine/phenolic resin composite powders with pine mass fractions of 20%, 30%, and 40%. Laser-sintered parts of pine/phenolic resin composites were prepared using a laser powder sintering rapid prototyping machine (CX-A200, Shanghai Tiannian Material Technology Co., Ltd., Shanghai, China), and the experimental procedure is shown in Figure 1.

**Figure 2:**
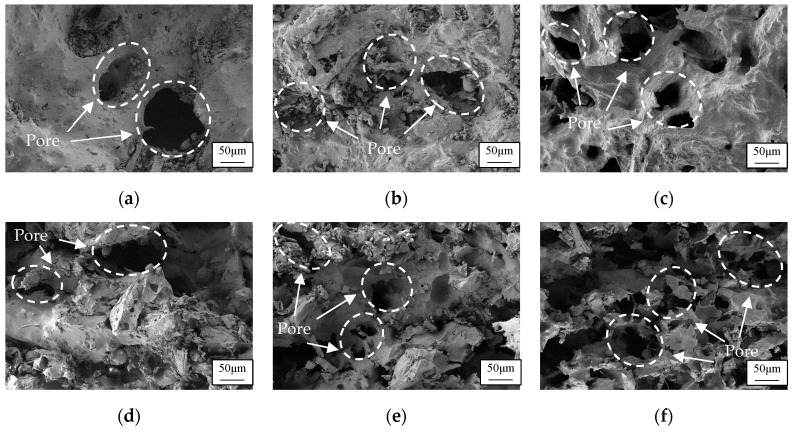
A 200-fold pore morphology on the surface and cross-section of sintered parts of pine/phenolic composite materials with different amounts of pine wood addition: (**a**) 20%, surface; (**b**) 30%, surface; (**c**) 40%, surface; (**d**) 20%, cross-section; (**e**) 30%, cross-section; (**f**) 40%, cross-section.

The surfaces and cross-sections of sintered specimens with 20%, 30%, and 40% pine wood mass ratios were magnified 200 times under an electron microscope. The microscopic pore morphology was observed. The porosity of the laser-sintered composite material, both on the surface and internally, increased proportionally with the pine powder content. Figure 2 illustrates the surface and cross-sectional scanning electron microscope images of sintered specimens with varying pine concentrations. The enhanced porosity of the sintered specimens provides an increased internal surface area, facilitating a more efficient conversion of sound energy into thermal energy and promoting sound wave scattering and reflection. These characteristics are advantageous for sound absorption. Consequently, the pine/phenolic resin composite powder with a 40% pine mass fraction, exhibiting the highest sintered porosity, was selected as the optimal laser sintering material for this investigation.

### 2.2. Bionic Structure Design

**Figure 3:**
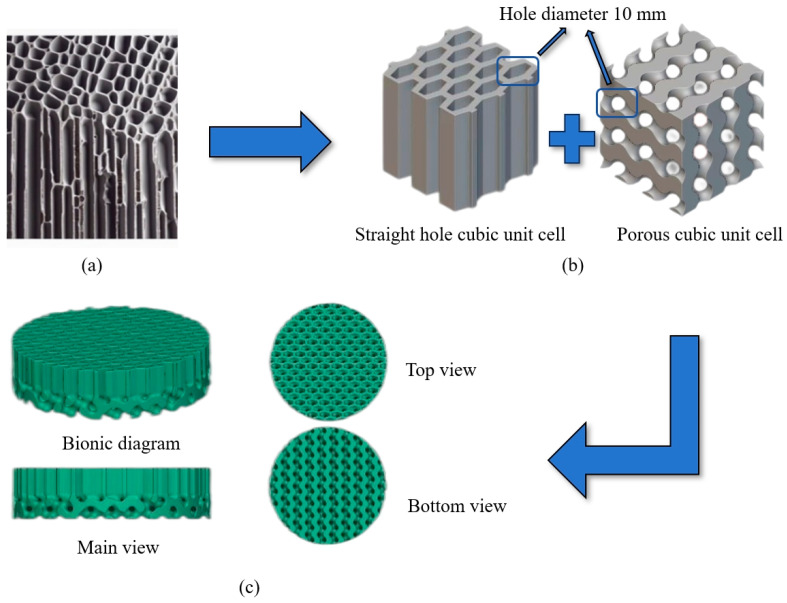
Evolution of the porous composite structure of biomimetic wood: (**a**) Microscopic diagram of the arrangement of wood tracheids; (**b**) Cell structure; (**c**) Biomimetic wood porous composite structure.

The acoustic properties of wood stem from its unique porous microstructure, which is formed by longitudinally arranged cells and fibers. As shown in Figure 3a, these cells and fibers form a network of micropores and cavities of varying sizes and shapes, giving the wood its irregular porous character. This natural structural complexity allows wood to effectively absorb sound waves over a wide frequency range [29]. In order to simulate this unique structure of wood, a biomimetic wood porous composite sound-absorbing structure was proposed. The structure consists of a straight-bore cubic unit cell and a porous cubic unit cell, as shown in Figure 3b. The combination of these two unit cells is designed to replicate the natural irregularities within the wood while maintaining the controllability and repeatability of the structure. The following tests were performed to investigate whether this bionic structure combines the high-frequency sound-absorbing properties of porous materials and the low-frequency sound-absorbing properties of perforated resonance structures. The unit cell size is uniformly set at 10 mm to ensure structural consistency and comparability. Figure 3c shows the porous composite structure of biomimetic wood designed based on the above principle.

### 2.3. Specimen Preparation

#### 2.3.1. Main Raw Materials and Equipment

(1) Materials:

- Pine flour: Light yellow powder (Shanghai Tiannian Material Technology Co., Ltd., Shanghai, China)
- Phenolic resin powder: Yellow powder, PF-2123 type (Jinan Dahui Chemical Technology Co., Ltd., Jinan, China)

(2) Equipment:

- Electric vibrating sieve machine: YCHH0301 (Yichang Huaheng Equipment Manufacturing Factory, Yichang, China)
- High-speed mixer: SHR-10A (Zhangjiagang Hongji Machinery Co., Ltd., Zhangjiagang, China)
- Constant temperature drying oven: DHG-9241 (Shanghai Jinghong Test Equipment Co., Ltd., Shanghai, China)
- Laser powder sintering rapid prototyping machine: CX-A200 (Harbin Free Intelligent Manufacturing Technology Development Co., Ltd., Harbin, China)
- Benchtop SEM: EM-30 Plus (Coxem Co., Ltd., Daejeon, Korea)

#### 2.3.2. Preparation of Experimental Samples

The pine powder was put into an electric vibrating sieve to screen out the powder with uniform particles of 160 mesh. It was then placed in a constant temperature oven at 100 °C for 12 h of drying. Subsequently, the pine powder was added to the phenolic resin after drying at 30 °C for 12 h, and the pine/phenolic resin composite powder was prepared according to the mass ratio of 40:60. The composite powder was then evenly mixed using a high-speed mixer.

**Figure 4:**
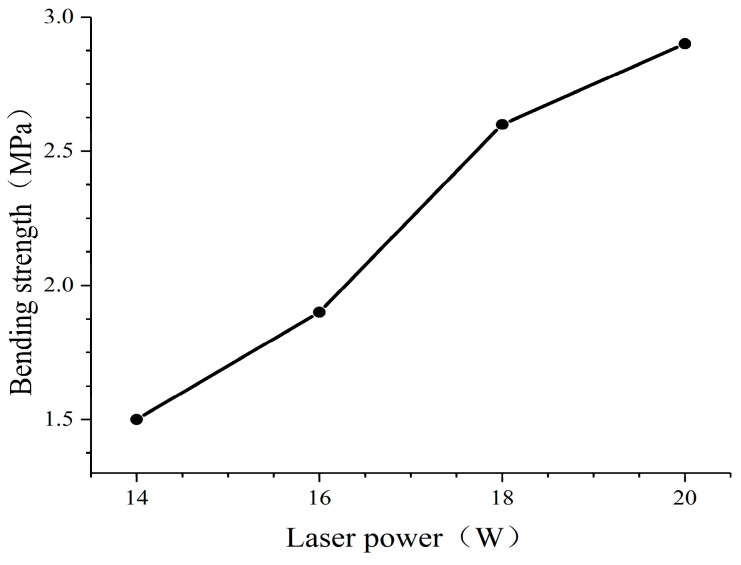
Bending strength of standard specimens under 14~20 W laser power.

**Figure 5:**
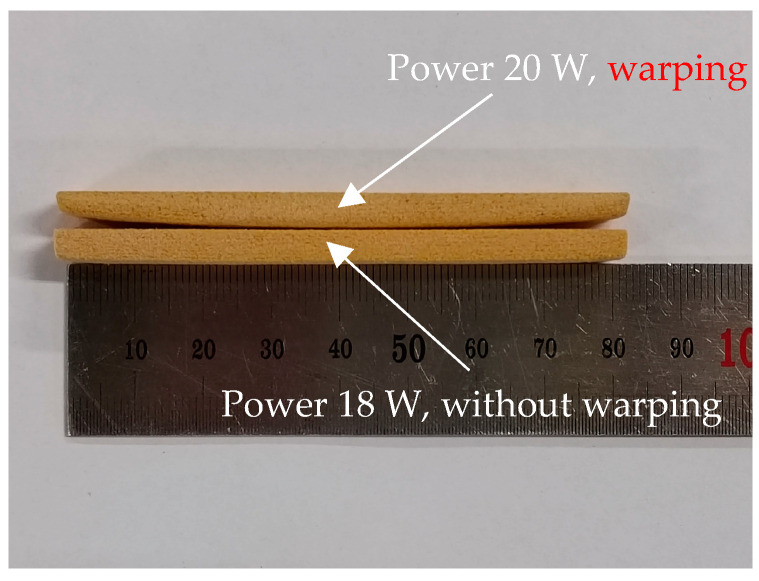
Warpage of sintered specimens.

The effect of different strength laser powers on the bending strength of the standard specimen was measured, and each specimen was repeated in three replicates with its average value taken as the final result. Figure 4 shows the effect of different laser powers on the forming strength of 40% pine/phenolic sintered parts. The laser sintering power was 14~20 W, and the bending strength of the standard specimens increased sequentially. However, when the sintering power is 20 W, there is warping at both ends of the specimen, and when the sintering power is 18 W, there is no obvious warping at both ends of the specimen, as shown in Figure 5.

**Figure 6:**
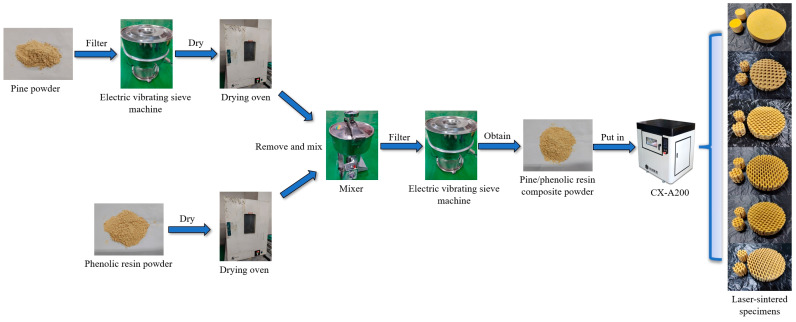
Step-by-step diagram of the preparation and experimental process of pine/phenolic resin composite powder.

The final experimental samples were prepared using the following parameters: laser power 18 W, scanning rate 1800 mm/s, preheating temperature 53 °C, scanning spacing 0.1 mm, and layer thickness 0.1 mm. The basic dimensions of the specimens are uniformly adopted: the diameter of the high-frequency test specimen is 30 mm, and the diameter of the low-frequency test specimen is 100 mm. In addition, the plate thickness is 25 mm. The preparation steps and laser sintering experimental process of pine wood/phenolic resin composite powder are shown in Figure 6.

### 2.4. Experimental Plan

This study adopts a systematic experimental design, divided into three main stages: first, six specimens labeled a to f are prepared, with descriptions provided in Table 1. The research investigates the effects of different porous composite structures on the sound absorption performance of laser-sintered specimens made from pine/phenolic resin in order to determine the optimal structural configuration.

Second, considering that the placement of the specimen in the impedance tube is different, the dissipation paths of the sound waves in the porous composite structure are also different. This explores the impact of the specimen placement method on the sound absorption effect to optimize the installation method in practical applications.

Finally, based on the experimental results, several representative laser-sintered specimens were selected for analysis. To investigate and analyze the effect of cavity thickness on the sound absorption performance characteristics of the specimens, the cavity thickness of the specimens was set to four gradients: 10 mm, 20 mm, 30 mm, and 40 mm.

All experiments used a single-factor experimental design to accurately quantify the impact of each factor on sound absorption performance. Three specimens were sintered under each type, and each specimen was measured three times, with the experimental results averaged to study their sound absorption performance.

### 2.5. Sound Absorption Testing Method

The impedance tube method is a measurement technique widely used in the field of acoustics, mainly for evaluating the acoustic properties of materials. Its core function is to calculate parameters such as acoustic impedance, absorption coefficient, and transmission loss of a material by measuring the characteristics of sound waves propagating and reflecting on the material surface. This method is of great importance in the fields of building acoustics, the automotive industry, aerospace, and environmental noise control. This study employed the impedance tube method for sound absorption testing, a technique widely recognized for its simplicity, operational convenience, broad frequency range, and high measurement accuracy [30]. The testing apparatus, provided by Beijing Prestige Company, consisted of a comprehensive impedance tube test system designed to evaluate sound absorption properties across various frequency ranges.

The experimental setup included two primary components: a 100 mm diameter impedance tube (SW422) for medium- and low-frequency testing and a 30 mm diameter impedance tube (SW477) for high-frequency analysis. To ensure precise data acquisition and processing, the system was complemented by auxiliary equipment, including a calibrator (CA111), a four-channel data collector (MC3242), a power amplifier (PA50), and VA-Lab noise and vibration test software.

The material to be measured is mounted at one end of the impedance tube. A loudspeaker is used to generate broadband sound waves. The sound pressure signal is collected by two microphones arranged along the wall of the tube. The transfer function method calculates the acoustic reflection coefficient of the material surface by analyzing the acoustic pressure signal between the two microphones and separating the incident and reflected waves. In turn, the sound absorption coefficient is deduced. Finally, the data are processed by analyzing software to generate the curve of sound absorption coefficient with frequency.

All tests strictly adhered to ISO 10534-2:1998 [31] (equivalent to National Standard GB/T 18696.2-2002 [32]), which outlines the transfer function method for measuring sound absorption coefficients and acoustic impedance in impedance tubes. The testing protocol covered a frequency range from 80 Hz to 6300 Hz, with sound absorption coefficients recorded at the center frequencies of 1/3 octave bands. This comprehensive approach ensured a thorough evaluation of the specimens’ acoustic properties across a wide spectrum of frequencies relevant to practical applications.

## 3. Result and Analysis

**Figure 7:**
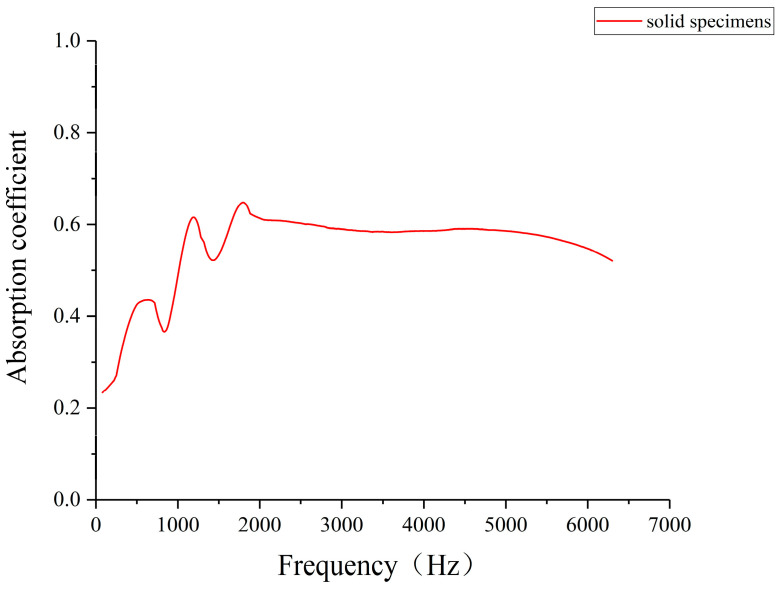
Sound absorption coefficient of solid laser-sintered specimens.

Impedance tube measurements of solid pine/phenolic laser-sintered specimens revealed significant sound absorption capabilities across the frequency spectrum (Figure 7). The specimens maintained a minimum absorption coefficient of 0.234 throughout, with notable performance in the mid-frequency range. Three distinct absorption peaks were observed at 636 Hz, 1194 Hz, and 1820 Hz, with coefficients of 0.435, 0.616, and 0.646, respectively, reaching a maximum at 1820 Hz. In the mid-high- to high-frequency range, the absorption coefficient exhibited relative stability with a slight downward trend. These findings suggest that pine/phenolic laser-sintered solid specimens possess favorable acoustic properties, particularly in mid-frequency applications, indicating their potential utility in various acoustic management scenarios.

The sound absorption mechanism of laser-sintered pine/phenolic resin composites can be attributed to their porous microstructure, which develops during the sintering process. These pores provide an extensive internal surface area that facilitates sound energy dissipation through air molecule friction and viscous motion, converting acoustic energy into thermal energy [33]. Additionally, the diverse pore sizes and geometries enable absorption across a broad frequency spectrum, with enhanced performance near pore resonance frequencies. This multi-scale porosity contributes to the material’s effective sound absorption capabilities, explaining the favorable acoustic properties observed in the solid laser-sintered specimens.

### 3.1. Effect of Different Porous Composite Structures on the Sound Absorption Performance of Laser-Sintered Specimens

**Figure 8:**
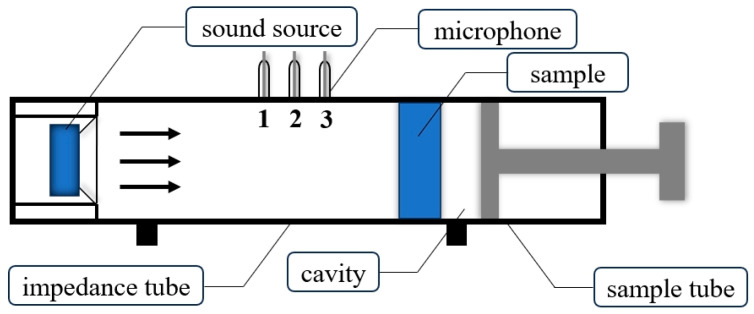
Schematic diagram of the impedance tube structure.

**Figure 9:**
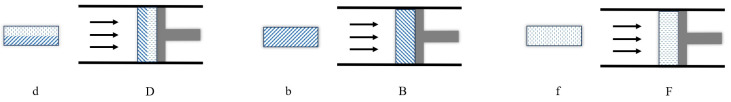
Schematic diagram 1 of the impedance tube testing. D: The d specimens with 1/2 each of straight and porous holes were placed inside the impedance tube, with the porous cells placed in front; B, F: Fully porous b specimens and fully straight hole f specimens were placed inside the impedance tube.

**Figure 10:**
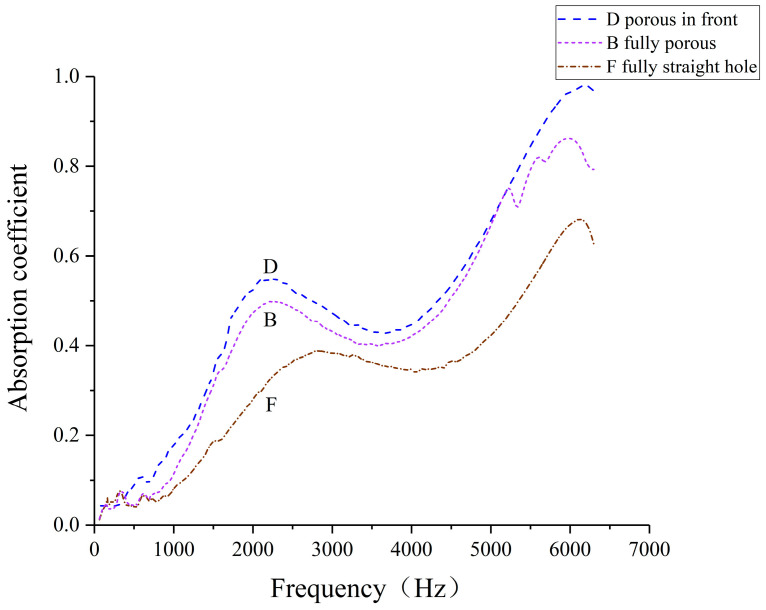
Sound absorption coefficient of three types of laser-sintered specimen testing.

The impedance tube testing configuration, illustrated in Figure 8, positions the sound source at the front end with a cavity thickness of 0 mm. Sound absorption coefficients were measured for three specimen types: fully porous (B), fully straight hole (F), and a hybrid structure (D) comprising equal proportions of porous and straight hole unit cells. Figure 9 and Figure 10 present the experimental conditions and resultant absorption characteristics for these specimens, providing a comparative analysis of their acoustic performance across various structural configurations.

Figure 10 demonstrates that the hybrid D-type specimen, featuring a porous structure at the front, outperforms both the fully porous (B-type) and fully straight hole (F-type) samples across nearly the entire frequency spectrum. This superior performance of the biomimetic wood porous composite structure over single-unit cell configurations can be attributed to the synergistic effect of diverse pore types, which enhances sound wave scattering and dissipation processes.

**Figure 11:**
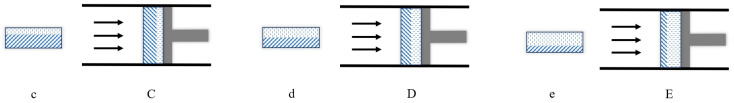
Schematic diagram 2 of the impedance tube testing. C: The porous unit cell of the c specimen inside the impedance tube is placed in front; D: The d specimens with 1/2 each of straight and porous holes were placed inside the impedance tube, with the porous cells placed in front; E: The porous unit cell of the e specimen inside the impedance tube is placed in front.

**Figure 12:**
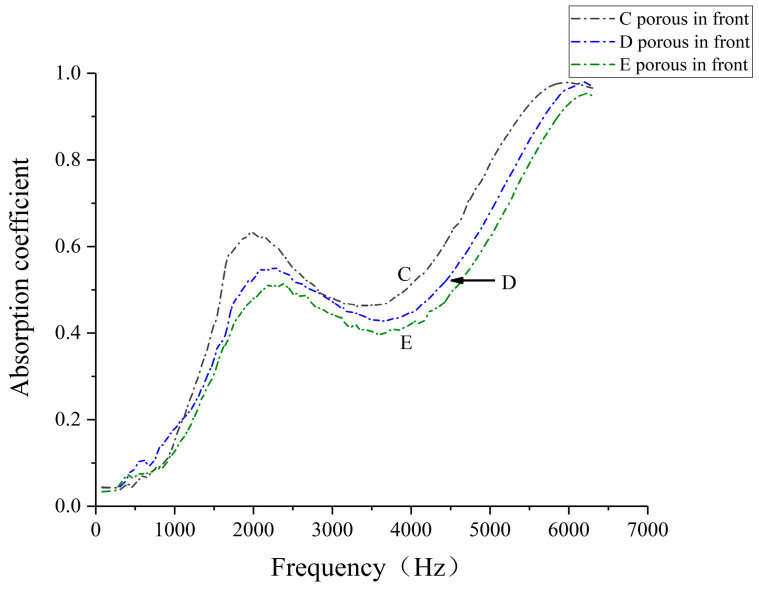
Sound absorption coefficient of laser-sintered specimens with different porous composite structures.

Further analysis focused on comparing the sound absorption coefficients of different porous combination structures: specimen C (38% straight hole cell, 62% porous cell), specimen E (62% straight hole cell, 38% porous cell), and specimen D (50% straight hole cell, 50% porous cell), all with porous fronts. The experimental conditions and results for these configurations are presented in Figure 11 and Figure 12, providing insights into the relationship between structural composition and sound absorption efficacy in biomimetic wood porous composites.

Figure 12 illustrates the sound absorption characteristics of specimens C, D, and E, all featuring porous fronts but with varying straight-hole-to-porous cell ratios. Specimen C demonstrates peak absorption in the mid-to-high frequency range, with maximum coefficients of 0.632 at 1992 Hz and 0.979 at 5958 Hz. Specimens D and E exhibit similar absorption patterns, with D showing peaks of 0.550 at 2112 Hz and 0.981 at 6186 Hz, while E peaks at 0.516 at 2350 Hz and 0.954 at 6212 Hz. Notably, in the 300–900 Hz range, specimen D outperforms both C and E in sound absorption. These results suggest that the proportion and arrangement of straight hole and porous cells significantly influence the acoustic performance across different frequency ranges, with the potential for optimizing absorption characteristics through structural design.

The experimental results demonstrate that the biomimetic wood porous composite structure exhibits superior sound absorption performance in the medium- to high-frequency range, characterized by two distinct absorption peaks. This contrasts with traditional perforated plate structures, which often suffer from limited high-frequency absorption and narrow effective frequency bands [34,35]. While single-layer microperforated plates typically offer effective absorption within a single octave, the biomimetic wood structure provides broader bandwidth absorption. This enhanced performance can be attributed to its unique multi-scale pore distribution and complex internal channel network, enabling effective absorption and dissipation of sound waves across a wider frequency spectrum [36]. Consequently, the biomimetic wood porous composite structure offers significant advantages over conventional designs, expanding its potential applications in acoustic management scenarios.

Figure 12 reveals similar sound absorption patterns for specimens C, D, and E, with type C demonstrating significantly higher absorption coefficients. This superior performance can be attributed to the increased thickness of the porous unit cell structure, which extends the sound wave propagation path and enhances energy dissipation [29]. Furthermore, the thicker porous structure promotes multiple reflections and scattering of sound waves, creating a complex internal sound field. This phenomenon, characterized by multiple reflections and resonances, prolongs the sound wave residence time within the material, thereby increasing opportunities for energy dissipation and ultimately improving the overall sound absorption efficacy of the structure.

### 3.2. Effect of Different Placements on the Acoustic Performance of Porous Composite Structures

**Figure 13:**
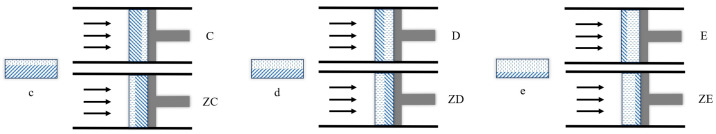
Schematic diagram 3 of impedance tube testing. The letter Z stands for straight pore unit cells placed in front; C, D, E: The porous unit cells of specimen c, specimen d, and specimen e in the impedance tube are placed in front; ZC, ZD, ZE: The straight pore cells of specimen c, specimen d, and specimen e in the impedance tube are placed in front.

**Figure 14:**
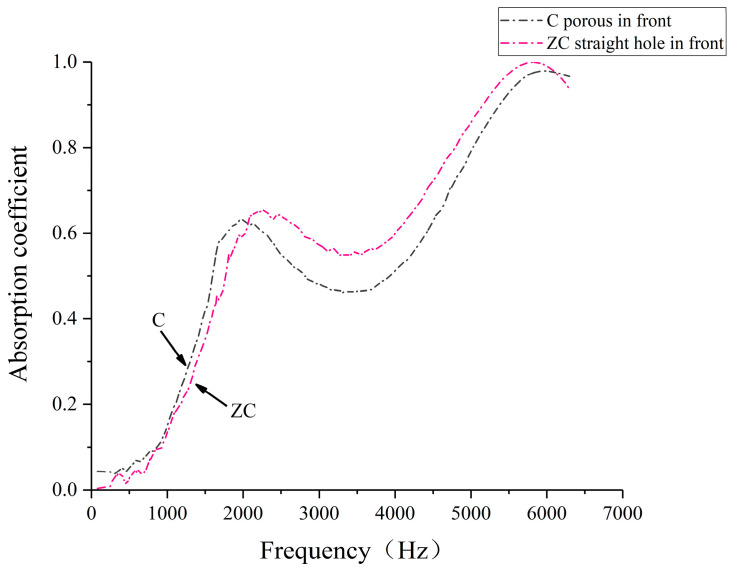
Sound absorption coefficient of the porous composite structures with different placements of the specimens.

**Figure 15:**
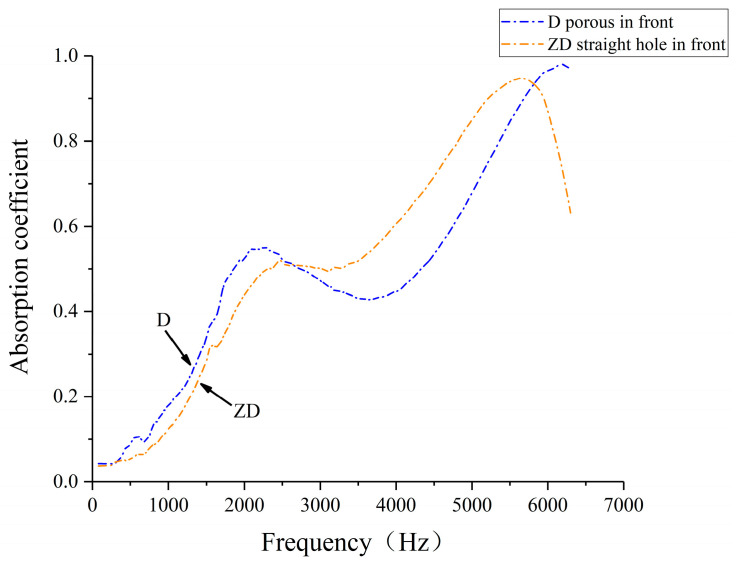
Sound absorption coefficient of the porous composite structures with different placements of the d specimens.

**Figure 16:**
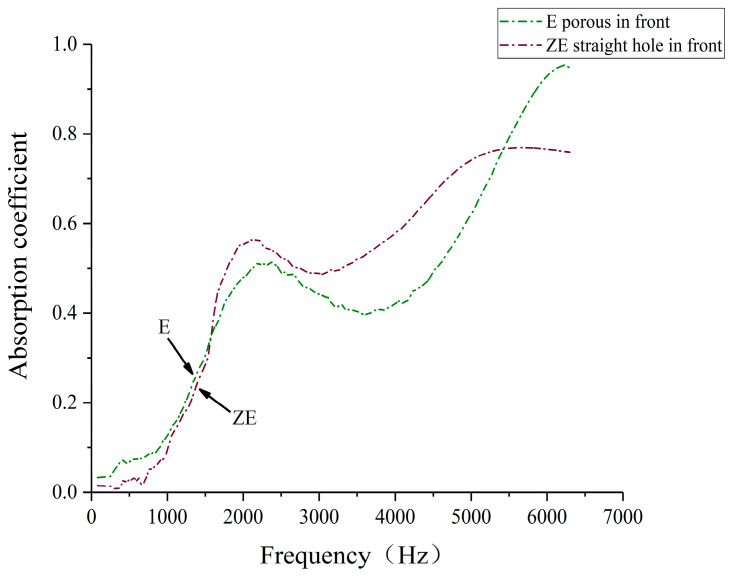
Sound absorption coefficient of the porous composite structures with different placements of the e specimens.

Considering the different ways of placing the specimens in the impedance tube and the different dissipation paths of the sound waves in the porous composite structure, the sound absorption coefficient of specimen C (38% straight hole unit cell, 62% porous unit cell), specimen D (50% straight hole unit cell, 50% porous unit cell), and specimen E (62% straight hole unit cell and 38% porous unit cell) was selected to test the sound absorption coefficient of the straight hole unit cell structure in the previous way. The sound absorption coefficient of laser-sintered specimens under different placement methods of porous in front and straight hole in front was compared and analyzed, and the test situation and results are shown in Figure 13, Figure 14, Figure 15 and Figure 16. The cavity thickness of this section of the experiment is 0 mm.

Figure 14, Figure 15 and Figure 16 illustrate the sound absorption characteristics of specimens ZC, ZD, and ZE, all featuring straight hole unit cell structures at the front. These specimens exhibit similar absorption patterns with two distinct peaks in the mid- and high-frequency ranges. The mid-frequency resonance peaks occur at 2268 Hz, 2474 Hz, and 2100 Hz for ZC, ZD, and ZE, respectively, with corresponding absorption coefficients of 0.654, 0.520, and 0.565. High-frequency resonance peaks are observed at 5812 Hz, 5656 Hz, and 5638 Hz, with maximum absorption coefficients of 0.999, 0.948, and 0.769 for ZC, ZD, and ZE, respectively. The similarity in absorption curves across these specimens suggests a consistent acoustic behavior for structures with straight hole frontal configurations, despite variations in their specific resonance frequencies and peak absorption values.

The data reveal that specimens with straight-pore frontal configurations exhibit superior sound absorption coefficients in the 2500–5500 Hz range compared to their porous-front counterparts. This enhanced performance in the mid- to high-frequency bands may be attributed to the improved initial sound wave dissipation offered by the regular straight hole structure. However, these specimens demonstrate narrower absorption bands and more rapid high-frequency coefficient decay. These findings suggest that the strategic arrangement of straight and porous cell structures in biomimetic wood porous composites can optimize sound absorption performance across different frequency ranges. Such tailored structural designs enable precise tuning of the material’s acoustic response, facilitating the achievement of broadband sound absorption objectives.

### 3.3. Effect of Different Cavity Thicknesses on Sound Absorption Performance of Laser-Sintered Specimens

To investigate the influence of cavity thickness on sound absorption performance, three laser-sintered pine/phenolic specimens were selected: a solid specimen A, specimen C (38% straight hole unit cells, 62% porous unit cells), and specimen D (50% straight hole unit cells, 50% porous unit cells). All specimens were configured with the porous unit cell structure at the front. Sound absorption tests were conducted with cavity thicknesses of 10 mm, 20 mm, 30 mm, and 40 mm for each specimen type. This experimental setup allows for a comprehensive analysis of how varying cavity thicknesses affect the acoustic properties of different biomimetic wood porous composite structures.

**Figure 17:**
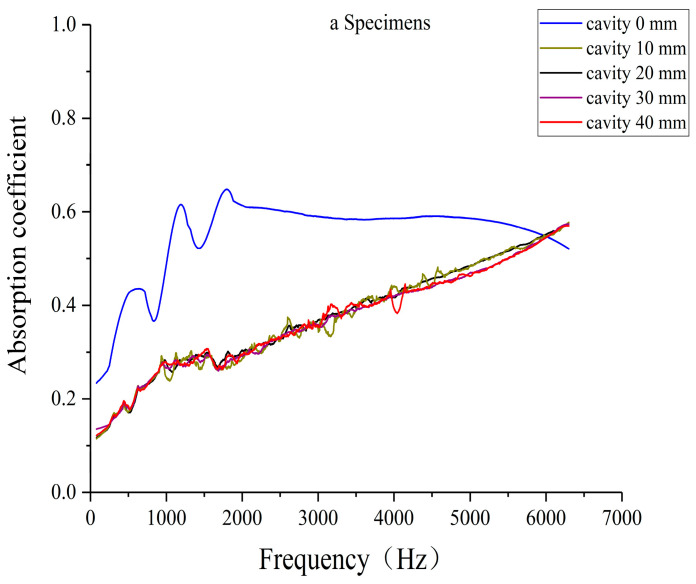
Absorption coefficient of solid specimens with different cavity thicknesses.

Figure 17 illustrates the sound absorption coefficients of solid specimens with varying cavity thicknesses. The introduction of cavities results in a marked decrease in sound absorption performance compared to specimens without cavities. For specimens with cavities, the sound absorption coefficient exhibits a gradual increase with frequency, and the absorption curves demonstrate a consistent pattern across different cavity thicknesses. This trend suggests that while the presence of cavities alters the acoustic properties of solid specimens, the relationship between frequency and absorption remains relatively uniform regardless of cavity thickness.

The introduction of cavities in pine/phenolic resin laser-sintered solid specimens results in consistent effects on sound absorption coefficients across various cavity thicknesses. While the resonance frequency shifts with cavity addition, the overall cavity effect remains minimal, potentially explaining the observed reduction in sound absorption coefficients. The uniformity of influence across different cavity thicknesses suggests that the initial presence of a cavity largely determines the fundamental sound wave reflection and scattering mechanisms within the structure. Consequently, further increases in cavity thickness do not significantly alter these established acoustic mechanisms, resulting in similar sound absorption behaviors regardless of specific cavity dimensions.

**Figure 18:**
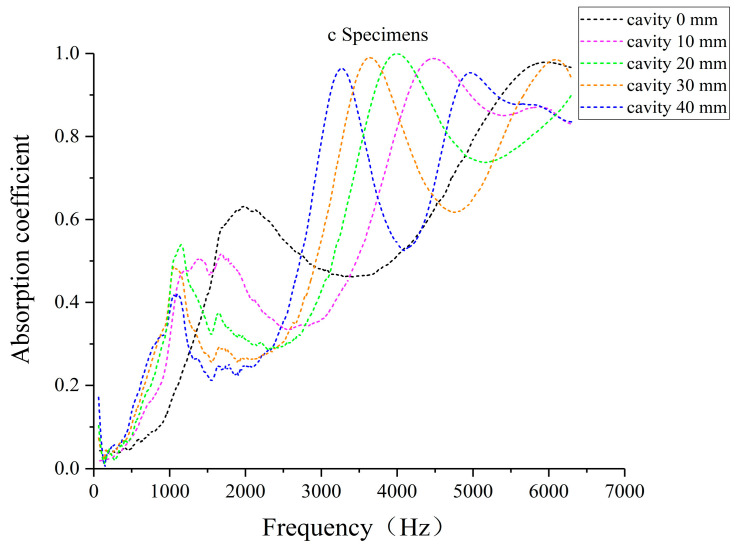
Sound absorption coefficient of the c specimen under different cavity thicknesses.

**Figure 19:**
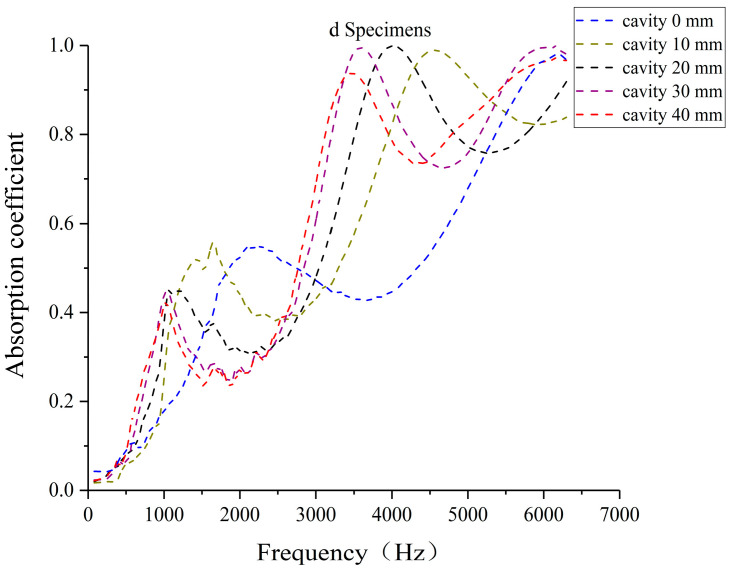
Sound absorption coefficient of the d specimen under different cavity thicknesses.

Figure 18 and Figure 19 demonstrate the sound absorption coefficients of specimens C and D with varying cavity thicknesses, featuring porous unit cell structures at the front. As cavity thickness increases from 10 mm to 40 mm, the absorption coefficient curves progressively shift toward lower frequencies. Mid-frequency resonance peaks migrate to lower frequencies with diminishing amplitudes, while high-frequency resonance peaks shift towards mid-high frequencies with relatively stable amplitudes. The introduction of cavities enhances sound absorption performance in the 500–1500 Hz and 3000–5000 Hz ranges. Notably, at cavity thicknesses of 30 mm and 40 mm, three distinct absorption peaks emerge in the low-, mid-high-, and high-frequency bands, aligning with findings by Tian Chunyu et al. [37]. This multi-band absorption capability expands the potential applications of these structures in acoustic engineering.

The observed shift of the sound absorption curves towards lower frequencies with increasing cavity thickness is attributed to a complex interplay of factors, including alterations in resonance frequency, mass-elastic system characteristics, sound wave propagation behavior, impedance matching, and acoustic cavity effects [37]. To assess the acoustic performance of the biomimetic wood porous composite structure, specimen C with a 30 mm cavity thickness was selected for comparative analysis against traditional perforated plates. Data from Table 2 reveal that the effective sound absorption bandwidth of the biomimetic structure extends to 5632 Hz, surpassing that of conventional perforated structures. This demonstrates the composite’s capacity for multi-band sound absorption and its notably broad effective bandwidth, highlighting its potential advantages in acoustic applications.

The biomimetic wood multi-porous composite structure emulates the intricate internal architecture of natural wood through the strategic arrangement of straight holes and porous cells. This design approach preserves the superior acoustic properties inherent to wood while offering enhanced flexibility for targeted frequency optimization. By leveraging bionic principles, this innovative structure presents a novel paradigm for developing high-performance acoustic materials. Its versatility and efficacy position it as a promising solution for diverse applications, spanning architectural acoustics, automotive interiors, and industrial noise mitigation, thus opening new avenues in advanced acoustic engineering.

## 4. Conclusions

This study aims to design a bionic wood porous composite structure and reveal the sound absorption performance of the laser-sintered porous composite structure of pine/phenolic resin. The specific conclusions are as follows:

(1) Laser sintering of pine/phenolic resin solid specimens results in the formation of abundant surface and internal pores, significantly enhancing their acoustic properties. This microstructural modification confers robust sound absorption capabilities, with the specimens maintaining a minimum absorption coefficient of 0.234 across the entire frequency spectrum.
(2) The biomimetic wood porous composite structure exhibits a broad sound absorption bandwidth, with particularly effective performance in the mid- to high-frequency range, characterized by dual absorption coefficient peaks. Despite relatively lower absorption at low frequencies, the structure maintains consistent absorption patterns across specimens with varying straight pore ratios (38%, 50%, 62%), showing a gradual decrease in absorption coefficients. Notably, configurations with straight pore cells at the front display a narrower absorption bandwidth but achieve higher absorption coefficients in the mid- to high-frequency range compared to their porous-front counterparts. This comparative analysis reveals the significant impact of structural arrangement on acoustic performance, offering insights for tailored design in specific frequency domains.
(3) The introduction of cavities in the composite structure yields complex effects on acoustic performance. Solid specimens with cavities exhibit reduced absorption coefficients compared to their non-cavity counterparts, with consistent absorption curves across various cavity thicknesses. In configurations where porous cells are positioned at the front, increasing cavity thickness progressively shifts the absorption coefficient curve toward lower frequencies. Notably, the presence of cavities enhances sound absorption in both mid-low (500–1500 Hz) and mid-high (3000–5000 Hz) frequency ranges. This nuanced impact of cavity design on frequency-specific absorption characteristics provides valuable insights for tailoring acoustic properties in targeted applications.
(4) The pine/phenolic resin laser-sintered porous composite structure demonstrates multi-band sound absorption capabilities and a broad effective frequency range, distinguishing it from conventional perforated plate absorbers. This unique acoustic profile expands its potential applications in sound absorption technology. By elucidating the structure–property relationships in these biomimetic composites, this study provides a robust theoretical foundation for the future development of laser-sintered materials and bionic structural designs in acoustic engineering. The findings offer valuable insights for optimizing material selection and structural configuration in advanced sound absorption applications.

## Figures and Tables

**Table 1:** Models and descriptions of six types of sintered specimens.

Type	Specimen Model	Specimen Structure Description
a	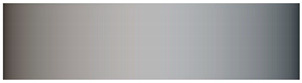	Solid pine/phenolic resin laser-sintered specimen (solid specimen), height 25 mm. (Side view example of the large impedance tube test piece, a~f diameter length 100 mm.)
b	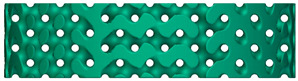	Laser-sintered specimens made entirely of porous cells (100% porous cells), 25 mm high.
c	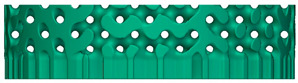	Laser-sintered specimens with a small proportion of straight hole crystal cell structure thickness (38% straight hole crystal cell, 62% porous crystal cell), straight hole height 9.5 mm, porous height 15.5 mm.
d	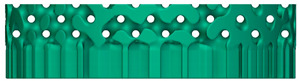	Laser-sintered specimens with the same thickness ratio of the straight hole crystal cell structure and porous crystal cell structure (50% straight hole cell, 50% porous cell), straight hole height 12.5 mm, porous height 12.5 mm.
e	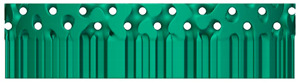	Laser-sintered specimens with a small proportion of porous cell structure thickness (straight cell 62%, porous cell 38%), straight cell height 15.5 mm, porous cell height 9.5 mm.
f	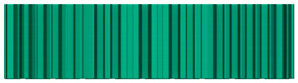	Laser-sintered specimens with 100% straight hole unit cells, height 25 mm.

**Table 2:** Comparison between the proposed sound-absorbing structure and previous works.

Compare Items	Limin Peng et al. [38]	Chunyu Tian [37]	This Study
Year	2018	2018	2024
Material type	Medium-density fiberboard	Metal	Wood–plastic composite material
Structural type	Wooden perforated panels	Perforated plates	Bionic porous combination structure
Test frequency range (Hz)	0~4000	0~6000	0~6300
Resonant frequency (Hz)	400	440, 1928, 3560, 5240	1062, 3640, 6098
Set whether the cavity is moved forward	Yes	Yes	Yes
Peak sound absorption coefficient	0.93	0.53, 0.70, 0.97, 0.97	0.49, 0.99, 0.99
Effective sound absorption bandwidth (Hz)	450	469, 209, 90, 68	5632

## Data Availability

The original contributions presented in the study are included in the article, further inquiries can be directed to the corresponding authors.
